# RiboaptDB: A Comprehensive Database of Ribozymes and Aptamers

**DOI:** 10.1186/1471-2105-7-S2-S6

**Published:** 2006-09-26

**Authors:** Venkata Thodima, Mehdi Pirooznia, Youping Deng

**Affiliations:** 1 Department of Biological Sciences, University of Southern Mississippi, Hattiesburg, Mississippi 39406, USA

## Abstract

**Background:**

Catalytic RNA molecules are called ribozymes. The aptamers are DNA or RNA molecules that have been selected from vast populations of random sequences, through a combinatorial approach known as SELEX. The selected oligo-nucleotide sequences (~200 bp in length) have the ability to recognize a broad range of specific ligands by forming binding pockets. These novel aptamer sequences can bind to nucleic acids, proteins or small organic and inorganic chemical compounds and have many potential uses in medicine and technology.

**Results:**

The comprehensive sequence information on aptamers and ribozymes that have been generated by *in vitro *selection methods are included in this RiboaptDB database. Such types of unnatural data generated by *in vitro *methods are not available in the public 'natural' sequence databases such as GenBank and EMBL. The amount of sequence data generated by *in vitro *selection experiments has been accumulating exponentially. There are 370 artificial ribozyme sequences and 3842 aptamer sequences in the total 4212 sequences from 423 citations in this RiboaptDB. We included general search feature, and individual feature wise search, user submission form for new data through online and also local BLAST search.

**Conclusion:**

This database, besides serving as a storehouse of sequences that may have diagnostic or therapeutic utility in medicine, provides valuable information for computational and theoretical biologists. The RiboaptDB is extremely useful for garnering information about *in vitro *selection experiments as a whole and for better understanding the distribution of functional nucleic acids in sequence space. The database is updated regularly and is publicly available at .

## Background

Until about 25 years ago, all known enzymes were proteins. But then it was discovered that some RNA molecules also have enzymatic property; that is, catalyze covalent changes in the structure of substrates (most of which are also RNA molecules) [1-3]. Catalytic RNA molecules are called ribozymes. Since the discovery of ribozymes that exist in living organisms, there has been a lot of interest in the study of new synthetic ribozymes made in the laboratory. First *Tang and Breaker *[4] lab isolated self-cleaving RNAs originating from random-sequence RNAs by using *in vitro *selection method. A large number of self-cleaving RNAs have been produced that have good enzymatic activity [5-7]. Some of the synthetic ribozymes that were produced had novel structures, while some were similar to the naturally occurring hammerhead ribozyme [2,8].

The aptamers are DNA or RNA molecules, possessing desirable affinity, selected by SELEX – Systemic Evolution of Ligands by Exponential enrichment method. This SELEX method is an *in vitro *iterative process that isolates binding aptamers from the random pool and amplifies each sequence by the polymerase chain reaction after each round of isolation [9-16]. The selected oligo-nucleotide sequences (~200 bp in length) have the ability to recognize specific ligands by forming binding pockets and can bind to nucleic acids, proteins or small organic, inorganic chemical compounds and even small organisms like viruses [17-25].

Aptamers are a promising class of compounds, both for target validation and therapy. As designer drugs, they exhibit high specificity, high affinity, and modifiable bioavailability [26-30]. The ability to generate inhibitors with such properties against a variety of target proteins will be invaluable as the human genome and proteome are deciphered [12,31-37].

The RiboaptDB is not only extremely useful both for identifying available aptamers and artificial ribozymes. It is also useful for acquiring information about *in vitro *selection experiments like the type of the nucleic acid, type of the target and conditions of the experiment as a whole and for better understanding the distribution of functional nucleic acids in the given sequence space. Like other types of sequences, the amount of sequences generated by *in vitro *selection experiments has been accumulating exponentially [10,14]. The sheer number and diversity of selection experiments has risen to the point where it is now essential to gather all the sequence data into a comprehensive, continuously updated database. The general sequence databases like GenBank, EMBL and DDBJ do not maintain the complete collection of artificial nucleic acid sequences like aptamer and ribozyme. Another database, 'Aptamer database' also contains lot of information on this type of data but not regularly updating with new data [38,39].

## Construction and Content

### Structure and implementation

**Figure 1:**
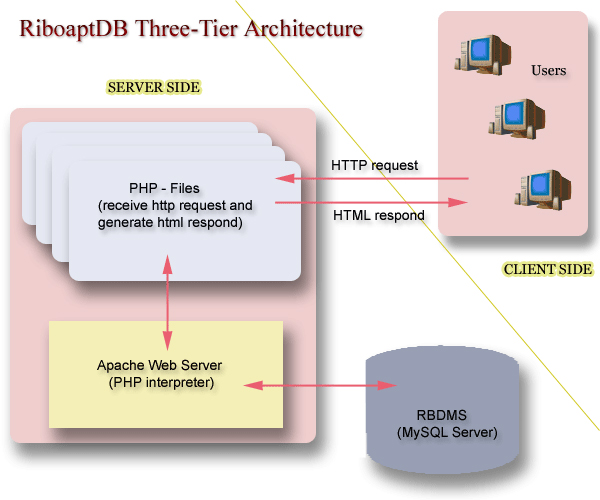
Schematic illustration of three tier architecture of the RiboaptDB.

**Figure 2:**
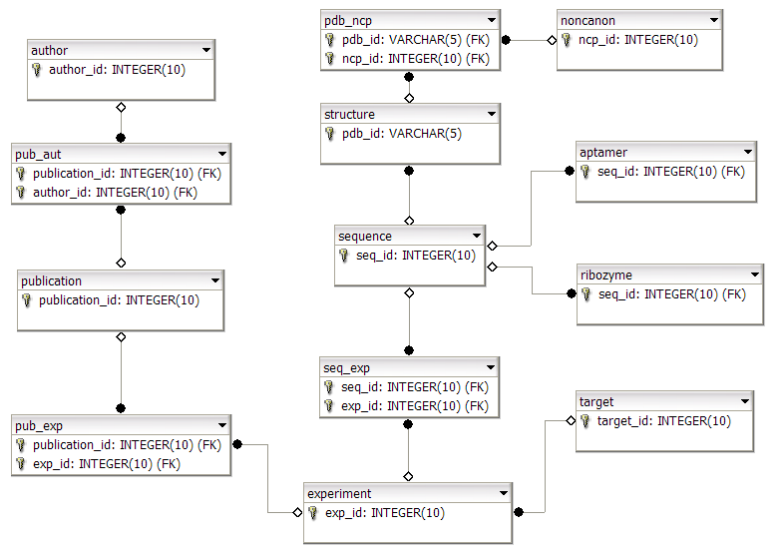
The schema and the dataflow of the RiboaptDB.

The design of the RiboaptDB database schema follows the three level schema architecture as shown in the Figure 1. The database has been implemented using open source relational database MySQL [40] server version 4.1. The user-friendly web interface has been developed by using PHP language [41]. The web server is an open source Apache web server version 2.0. The figure 2 shows the Entity-Relationship (ER) schema of the database.

The "sequence" table is the key table in the database to which all other tables are related directly or indirectly. This table contains the sequence ID and relates directly with its child tables, "aptamer" and "ribozyme", which contains the corresponding sequence information. The other important tables in the database are "publication" and "experiment" which store the citation information like title, journal name, authors, pubmed ID and experiment details like template type and experiment conditions respectively. The target specific information, the target name and its category ('organic', 'inorganic', 'nucleic', 'peptide', 'protein' and 'other') obtained from the "target" table. If any information about non-canonical base pair is available, it can be retrieved through the "non-canonical" table.

### Content

RiboaptDB is relatively small database but is, nonetheless, essentially complete. The data was sourced from a previous compilation and exhaustive searching of the primary literature. The current size of the database 4212 sequences from 423 citations.

In this, there are 370 artificial ribozyme sequences and 3842 aptamer sequences in the total 4212 sequences. The database is updated every month as new literature comes on aptamers and artificial ribozyme seqences. The intial collection of data is done through searching the NCBI-Pubmed for the literature with keywords like 'artificial ribozymes', 'ribozyme', 'aptamers', 'SELEX' etc. The usefulness of a database is governed by the accuracy of the data it contains. The data in this database is compiled manually from previous published, peer-reviewed articles, and verified.

## Utility and Discussion

**Figure 3:**
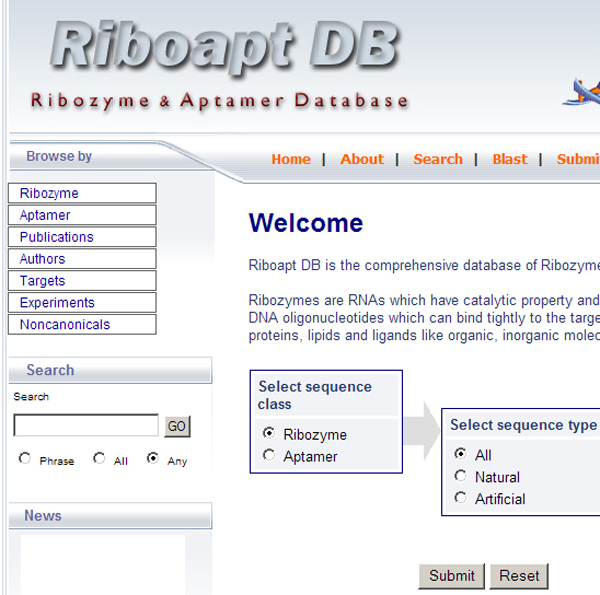
Screenshot of the homepage of the RiboaptDB, an user friendly interface for the database.

RiboaptDB provides users with an easy-to-use web interface with flexibility to select either ribozyme or aptamer sequences to browse the corresponding information. Beginning at the welcome page (Figure 3), the user can navigate via the top menu or the browse database tables on the side menu. A brief description about the navigation is given below.

### Search

The general complete search option provides an interface for a variety number of queries to the database. It can be used to search the database for sequence, experiment, target, author, publication and non-canonical along with either ribozyme or aptamer or both and also either natural or artificial type of sequences.

### Local Blast

**Figure 4:**
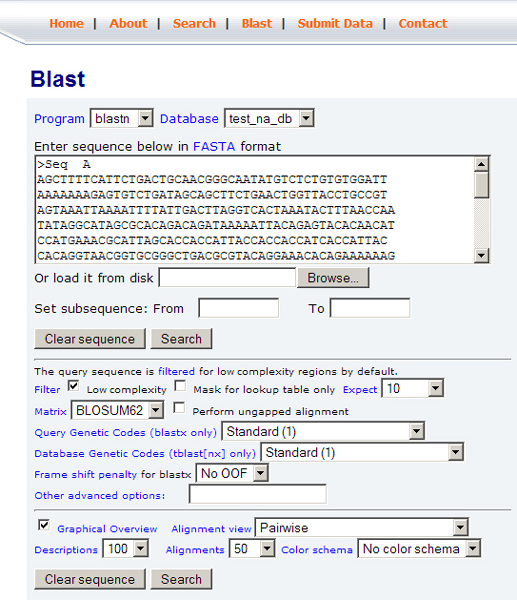
Screenshot of the local BLAST page with default options.

**Figure 5:**
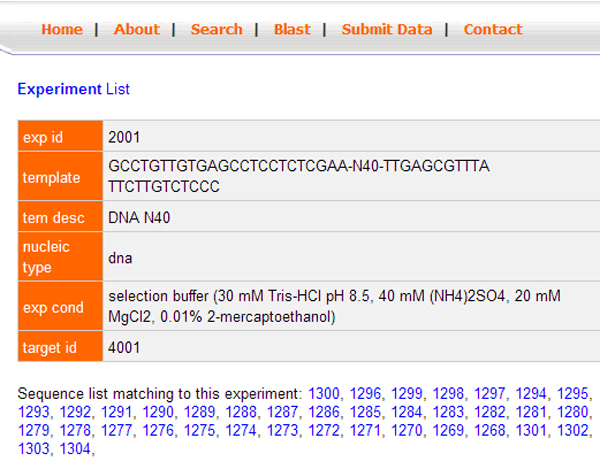
View of experiment details.

The local Blast option can be used to do blast search against the local archived data to perform sequence-similarity searches using the BLAST family of programs (Figure 4). This will useful to user to know the most similar sequences to the submitted sequences and also useful to know further information about its target and experiment details (Figure 5).

### Submit data

**Figure 6:**
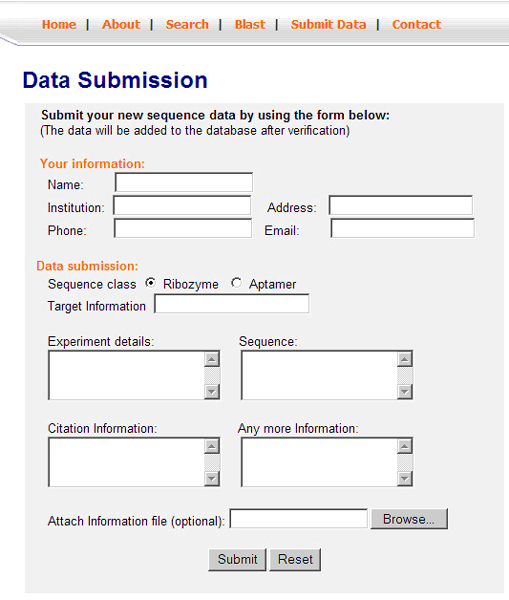
View of the data submission form by the user.

It facilitates online sequence submission to the database. It allows users to fill in a form containing new sequence information along with user details (Figure 6). Related information can also be submitted through uploading a text file. The data which is then saved into a directory on the server side and an email is sent automatically to the curator who then checks the data to make sure there are no errors and then the information is loaded into the database automatically.

Alternate to the general search option on top menu, there is a search option on the side menu on the home page to search the whole database on a specific keyword. Also, specific table search is available on side menu of each related pages. The user can also retrieve the selected sequences into a text file for further studies.

The idea behind the combining of ribozymes and aptamers data into one database is, increasing the chance of generating ribozymes with modified and novel properties [26]. One example is combining both the 'target identification' of aptamer and 'catalytic activity' of ribozymes into a commercial 'riboswitch' application [42-45].

### Future Perspectives

RiboaptDB project is young. With respect to future work, the database needs to be maintained and developed regularly, ensuring our links to external databases remain up to date and newly published data is added. Initially, as with all databases, random errors will have occurred due to human error during the data accumulation or will be extant within the original experimental data. The database will be assessed for errors and inconsistencies, thus maintaining, as far as possible, the overall veracity of our data.

## Conclusion

The goal of RiboaptDB constructors was the collection of all ribozyme and aptamer sequences that have appeared to date and their detailed and correct annotation. The ease of access to the data is of great importance and the bespoke search system and the inclusion of a BLAST search greatly facilitates this. The better the organisation of the data, the easier the work will be for researchers dealing with aptamers and ribozymes.

## Availability and Requirements

RiboaptDB was created and is maintained in the Department of Biological Sciences at the University of Southern Mississippi. It is publicly available at the .

## List of abbreviations

SELEX: Systemic Evolution of Ligands by Exponential enrichment

BLAST: Basic Local Alignment Search Tool

EMBL: European Molecular Biology Laboratory

DDBJ: DNA Data Bank of Japan

## Authors' contributions

VT and MP both participated in the design and implementation of the study of the database. VT conceived of the study, worked on identification of relevant data sources, comprehensive data annotation and drafted the manuscript. MP carried out the design and development of the user interface of the database. YD coordinated and directed the project and revised the manuscript. All authors have read and approved the final manuscript.
